# Force‐frequency relationship and early relaxation kinetics are preserved upon sarcoplasmic blockade in human myocardium

**DOI:** 10.14814/phy2.13898

**Published:** 2018-10-22

**Authors:** Jae‐Hoon Chung, Benjamin D. Canan, Bryan A. Whitson, Ahmet Kilic, Paul M. L. Janssen

**Affiliations:** ^1^ Department of Physiology and Cell Biology The Ohio State University Wexner Medical Center Columbus Ohio; ^2^ Dorothy M. Davis Heart and Lung Research Institute The Ohio State University Wexner Medical Center Columbus Ohio; ^3^ Medical Scientist Training Program and Biomedical Sciences Graduate Program The Ohio State University Wexner Medical Center Columbus Ohio; ^4^ Division of Cardiac Surgery Department of Surgery The Ohio State University Wexner Medical Center Columbus Ohio; ^5^ Department of Internal Medicine The Ohio State University Wexner Medical Center Columbus Ohio

**Keywords:** Contraction, EC coupling, heart failure, ryanodine receptor, SERCA, trabeculae

## Abstract

In this study, we investigated the quantitative and qualitative role of the sarcoplasmic reticulum (SR) in the regulation of the force‐frequency relationship (FFR). We blocked the function of SR with cyclopiazonic acid (CPA) and ryanodine and measured twitch kinetics and developed force at various stimulation frequencies in nonfailing and failing intact human right ventricular trabeculae. We found that developed forces are only slightly reduced upon SR blockade, while the positive FFR in nonfailing trabeculae and negative FFR in failing trabeculae were both preserved. The contraction kinetics (dF/dt, dF/dt/F, and time to peak), however, were significantly slower at all frequencies tested. Kinetics of first 50% of relaxation (RT50) was not affected by SR blockade. Kinetics of entire relaxation process (RT90) was overall slower at low frequencies, but not at high frequencies. From our findings, we conclude that the SR is not essential for FFR, and its role in regulation of FFR lies mostly in contraction kinetics. Unlike small rodents, human myocardium contractile function is near‐normal in absence of a functional SR with little changes in contractile force, and with preservation with the main regulation of FFR.

## Introduction

The heart increases its contractile capacity mainly via three mechanisms: (1) Frank‐Starling Mechanism, (2) frequency‐dependent activation, and (3) beta‐adrenergic activation. These three mechanisms work in synergy to allow for modulation of contraction and relaxation capacities in myocardium. Frequency‐dependent acceleration of relaxation (FDAR) is a physiological phenomenon that allows the heart muscle to relax faster as the pacing frequency is increased (Varian and Janssen 2007). As the heart rate increases due to more frequent electrical signal generation from the sinoatrial (SA) node, the cardiomyocytes must accelerate their contraction and relaxation kinetics in order to accommodate sufficient filling time with a decreased cycle time. If ventricular cardiomyocytes cannot relax quickly enough, the ventricles cannot fill with sufficient amount of blood before the next beat, which then in turn negatively impacts cardiac output. Multiple labs have reported slower relaxation kinetics in heart failure, but the exact mechanism of the dysfunction has not been elucidated (Gwathmey et al. 1991; Hasenfuss et al. 1992, 1994a; Chaudhary et al. 2004; Rossman et al. 2004). There are multiple factors that affect the relaxation kinetics in a cardiomyocyte, including (1) calcium dissociation from troponin C (TnC), (2) cross‐bridge cycling, and (3) calcium sequestration into sarcoplasmic reticulum (SR) and extrusion from the myocyte (Biesiadecki et al. 2014). Calcium sequestration into the SR is mediated by sarcoplasmic reticulum ATPase (SERCA). The expression levels of SERCA have been reported to be decreased, on average, with heart failure to result in increased diastolic intracellular calcium concentration (Hasenfuss et al. 1994b; Zarain‐Herzberg et al. 1996). The SR has been shown to play an important role in frequency‐dependent regulation of contraction and relaxation (Meyer et al. 1999; Bluhm et al. 2000). To quantify this role, we studied the force‐frequency relationship (FFR) in presence and absence of a functional SR. To abolish SR calcium cycling, we have used cyclopiazonic acid (CPA) and ryanodine in nonfailing and failing intact right ventricular trabeculae. CPA and ryanodine lead to a depletion of SR calcium, and it has been used in rat, rabbit, dog, and human and cardiac muscle and myocytes (Kentish and Wrzosek 1998; Maier et al. 1998; Hasenfuss et al. 2002; von Lewinski et al. 2004; Monasky and Janssen 2009; Torres et al. 2013; Kistamas et al. 2015). Previous studies showed that small rodents such as rats rely heavily on the SR for the sequestration of intracellular calcium to a much greater extent, as they sequester ~93% of intracellular calcium via the SR. Larger mammals, such as the rabbit, cycle ~70% of their intracellular calcium via the SR and ~30% via sodium calcium exchanger and sarcolemmal calcium channel (Maier et al. 1998; Monasky and Janssen 2009), and show a more preserved contractile profile. In this study, we investigate and quantify the quantitative and qualitative contribution of the SR to the FFR, as well as the impact of disease on this process.

## Methods

### Procurement of human hearts

All experiments were approved by the Institutional Review Board (IRB) at The Ohio State University Wexner Medical Center. Nonfailing hearts (*n* = 6) were obtained from the LifeLine of Ohio Organ Procurement, and failing hearts (*n* = 11) were obtained from patients undergoing cardiac transplantation at the Ohio State University Wexner Medical Center. Patient characteristics and information about the hearts are given in Tables 1 and 2. Nonfailing hearts were included in the study if they were considered for transplantation based on the condition of the heart, but not transplanted because of technical reasons (size‐mismatch, presence of infection, or minor defect that would have not impacted on cardiac contractile ability). Immediately after the hearts were explanted, they were coronary‐perfused with ice‐cold cardioplegic solution containing: 110 mmol/L NaCl, 0.5 mmol/L CaCl_2_, 16 mmol/L KCl, 16 mmol/L MgCl_2_ 6H_2_O, and 10 mmol/L NaHCO_3_ at pH 7.4. In ~20 min after explantation, the hearts were transported to the laboratory for experiments (Milani‐Nejad et al. 2015; Elnakish et al. 2017).

**Table 1:** Characteristics of nonfailing hearts

	Race	Sex	Age	Heart weight (g)	Cause of death	LVEF (%)
872295	Caucasian	Male	56	539	Anoxia, cardiac arrest	40
179155	Caucasian	Female	50	350	ICH/Stroke, cardiac arrest	ECHO not done
559324	African American	Female	59	583	Anoxia, cardiac arrest	65
799415	Caucasian	Female	45	404	Anoxia, cardiac arrest, drug intoxication	ECHO not done
731397	Caucasian	Female	54	548	ICH/Stroke	65
652357	Caucasian	Female	40	310	Anoxia	65

LVEF, left ventricular ejection fraction; ICH, intracerebral hemorrhage.

**Table 2:** Characteristics of failing hearts

	Race	Sex	Age	Heart Wt.(g)	Etiology	LVAD	Arrhythmia?
948463	Caucasian	Male	69	645	NICM	X	
465645	Caucasian	Male	59	972	NICM		
682814	Caucasian	Male	46	556	NICM	(HVAD)	
235847	Caucasian	Male	65	506	NICM	X	
132642	Caucasian	Male	48	839	ICM		
421856	Caucasian	Female	55	293	NICM		X
445852	Caucasian	Female	58	568	NICM	X	X
492613	African‐American	Female	42	463	NICM		X
897154	African‐American	Female	53	428	NICM	X	X
437918	Caucasian	Male	40	731	NICM	(HVAD)	X
916288	Caucasian	Male	56	657	NICM		X

All had EF < 25%. ICM, ischemic cardiomyopathy. NICM, nonischemic cardiomyopathy. LVAD, Left ventricular assistive device. HVAD, HeartWare ventricular assistive device.

### Isolation of intact cardiac trabeculae

In the laboratory, the ventricles were placed in ice‐cold modified Krebs‐Henseleit (KH) solution previously bubbled with 95% O_2_–5% CO_2_ containing: 137 mmol/L NaCl, 5 mmol/L KCl, 0.25 mmol/L CaCl_2_, 20 mmol/L NaHCO_3_, 1.2 mmol/L NaH_2_PO_4_, 1.2 mmol/L MgSO_4_, 10 mmol/L dextrose, and 20 mmol/L 2,3‐butanedione monoxime (BDM) at pH 7.4. Thin, linear, free‐hanging trabeculae were dissected from the right ventricle and stored at 4°C. Trabeculae experiments were performed within ~12 h after procurement of hearts. Trabeculae were mounted in a custom‐made setup that contains a force transducer and length manipulator and are superfused with Krebs‐Henseleit solution bubbled with 95% O_2_‐5% CO_2_ containing: 137 mmol/L NaCl, 5 mmol/L KCl, 0.25 mmol/L CaCl_2_, 20 mmol/L NaHCO_3_, 1.2 mmol/L NaH_2_PO_4_, 1.2 mmol/L MgSO_4_, and 10 mmol/L dextrose. The concentration of CaCl_2_ was gradually increased to 2 mmol/L over ~20 min. Then trabeculae were stretched via length manipulator to their optimal lengths at which the increase in developed force is equal to the increase in resting force, and their dimensions (length, width, and thickness) were measured via inverted microscope. At this point, we excluded trabeculae with <1 mN/mm^2^ developed force because of low signal‐to‐noise ratio and made sure that force tracings were smooth for appropriate kinetics analysis.

### Twitch kinetics measurement and superfusion with CPA/ryanodine

Trabeculae were stabilized for ~15 min, and twitch kinetics were measured after exposing the trabeculae to 0.5, 1, 1.5, 2, 2.5, and 3 Hz stimulation frequencies for ~3 min each (nonfailing *n* = 4 and failing *n* = 5, 1 trabecula from 1 heart). In dark room, 10 *μ*mol/L CPA (Sigma‐Aldrich, CAS 18172‐33‐3) and 1 *μ*mol/L ryanodine (Sigma‐Aldrich, CAS 15662‐33‐6) were added to the superfusate and the trabeculae were incubated for 20 min. Following 20‐min incubation, twitch kinetics were measured at 0.5, 1, 1.5, 2, 2.5, and 3 Hz frequencies.

### Effect of SR blockade on rapid cooling contractures on human right ventricular trabeculae

To verify SR block occurred, in a subset of experiments rapid cooling contractures were performed before and after application of CPA and ryanodine in the same human right ventricular trabecula (*n* = 4, from two hearts). Trabeculae were stimulated at 0.5 Hz and temperature was changed from 37°C to ~1°C within 1 sec as previously described (Pieske et al. 1999; Hasenfuss et al. 2002; Monasky and Janssen 2009).

### Data and statistical analysis

Twitch kinetics were analyzed with custom‐made Labview program. Specific forces were determined by dividing total force by the cross‐sectional area of the muscle. Statistical analysis was performed via analysis of variance (ANOVA), or by two‐way ANOVA (with or without repeated measures), where applicable, followed by appropriate post‐hoc tests. Statistical significance was set at *P *<* *0.05 two‐tailed, and data in figures are shown as mean ± SEM.

## Results

### Effect of SR blockade on rapid cooling contracture

**Figure 1:**
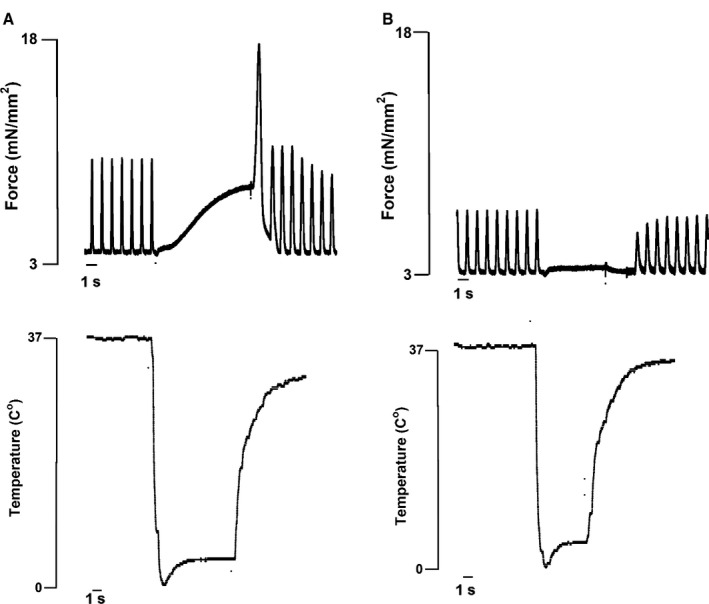
Rapid‐cooling contracture on a human nonfailing right ventricular trabecula stimulated at 0.5 Hz. (A) Rapid‐cooling contracture before SR blockade. (B) Rapid‐cooling contracture after SR blockade on the same trabecula shows depletion of SR calcium. SR, sarcoplasmic reticulum.

Figure 1A shows a rapid cooling contracture in a nonfailing trabeculae before application of CPA and ryanodine. After application of and CPA and ryanodine to the same trabecula, no active developed force was observed, indicative of successful SR calcium handling blockade (Fig. 1B).

### Cardiac contraction and relaxation can be sustained with SR blockade

**Figure 2:**
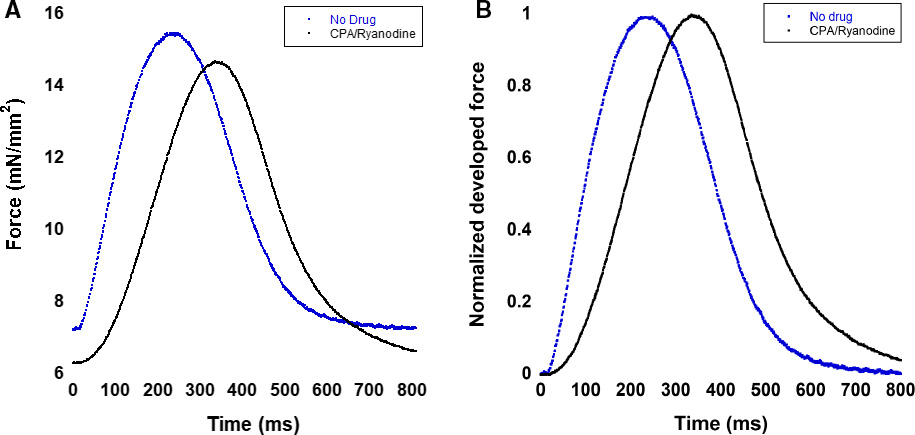
Example of a nonfailing trabeculae contracting at 1 Hz with and without SR blockade. (A) Twitches before and after SR blockade. Upon SR blockade, contraction kinetics are slower. (B) Twitches normalized to their maximal force before and after SR blockade. The slowing of contraction kinetics is seen more clearly, and relaxation kinetics do not change significantly. SR, sarcoplasmic reticulum.

At baseline (stimulated at 1 Hz), nonfailing and failing trabeculae were able to sustain twitches without a functional SR (Fig. 2A). In this typical example, upon SR blockade, the trabecula was able to generate force although the contraction kinetics were slower (Fig. 2A and B).

### SR blockade does not alter force‐frequency relationship

**Figure 3:**
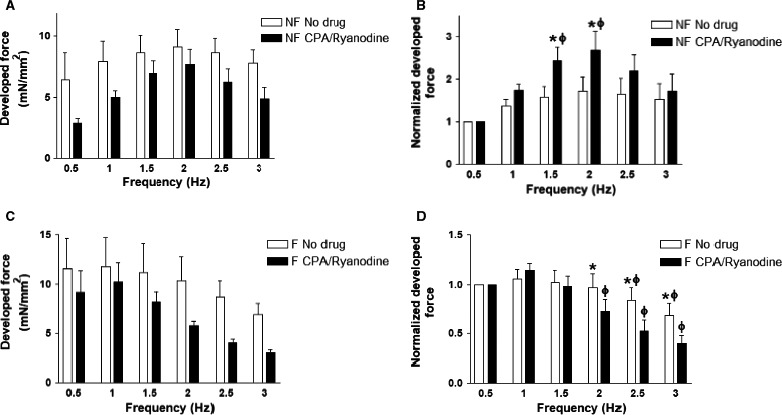
Developed force in nonfailing and failing trabeculae with and without SR blockade. (A) Developed force in nonfailing trabeculae (*n* = 4) decreased overall upon SR blockade, but statistical significance was not reached at specific frequencies. B) Normalized developed force in nonfailing trabeculae (*n* = 4) increased overall upon SR blockade with statistical significance at 1.5 and 2 Hz. (C) Developed force in failing trabeculae (*n* = 5) decreased overall upon SR blockade, but statistical significance was not reached at specific frequencies. (D) Normalized developed force in failing trabeculae (*n* = 5) decreased overall upon SR blockade with statistical significance at 2, 2.5, and 3 Hz. * indicates *P* < 0.05 before versus after SR blockade after two‐way ANOVA with Bonferroni post‐hoc test. Φ indicates *P* < 0.05 versus corresponding 0.5 Hz parameter in the same group after two‐way ANOVA with Bonferroni post‐hoc test. SR, sarcoplasmic reticulum.

Before SR blockade, nonfailing trabeculae typically displayed a positive FFR, and failing trabeculae a negative FFR. Upon SR blockade, the developed force in nonfailing and failing trabeculae decreased overall, but with no statistical significance at specific frequencies (ANOVA, *P < *0.05) (Fig. 3A and C). We normalized developed forces to 0.5 Hz to assess the changes in developed forces at higher frequencies and determine FFR. Interestingly, SR blockade resulted in more positive FFR curves in nonfailing trabeculae, especially at 1.5 and 2 Hz (ANOVA, *P < *0.05; *P < *0.05). SR blockade resulted in more negative FFR in failing trabeculae, especially at 2, 2.5, and 3 Hz (ANOVA, *P < *0.05; *P < *0.05). However, overall trends of positive FFR for nonfailing trabeculae and statistically significant negative FFR in failing trabeculae (ANOVA, *P *<* *0.05) were maintained after application of CPA and ryanodine. (Fig. 3B and D).

### SR blockade results in an increase in diastolic force at high frequencies

**Figure 4:**
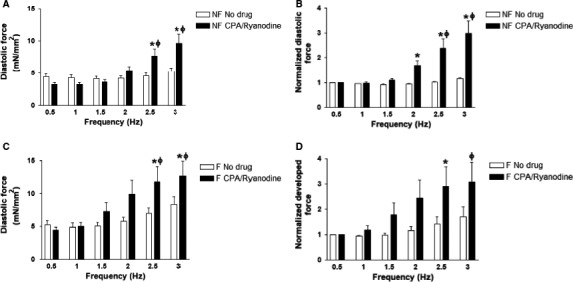
Diastolic force in nonfailing and failing trabeculae with and without SR blockade. (A) Diastolic force in nonfailing trabeculae (*n* = 4) increased overall upon SR blockade with statistical significance at 2.5 and 3 Hz. (B) Diastolic force in failing trabeculae (*n* = 5) increased overall upon SR blockade with statistical significance at 2.5 and 3 Hz. (C) Normalized diastolic force in nonfailing trabeculae (*n* = 4) increased overall upon SR blockade with statistical significance at 2, 2.5, and 3 Hz. (D) Normalized diastolic force in failing trabeculae (*n* = 5) increased overall upon SR blockade with statistical significance at 2.5 Hz. * indicates *P *<* *0.05 before versus after SR blockade after two‐way ANOVA with Bonferroni post‐hoc test. Φ indicates *P* < 0.05 versus corresponding 0.5 Hz parameter in the same group after Two‐way ANOVA with Bonferroni post‐hoc test. SR, sarcoplasmic reticulum.

Nonfailing and failing trabeculae exhibited an overall increase in diastolic force upon SR blockade, especially at 2.5 and 3 Hz (ANOVA, *P *<* *0.05; *P < *0.05) (Fig. 4A and C). Compared to 0.5 Hz, nonfailing trabeculae had an increase in diastolic force at 2.5 and 3 Hz after SR blockade (*P < *0.05). Failing trabeculae had the same pattern of having increased diastolic force compared to 0.5 Hz at 2.5 Hz and 3 Hz (*P *<* *0.05). We normalized diastolic forces to those at 0.5 Hz to more closely examine the change in diastolic force at various frequencies and found that normalized diastolic force was increased after SR blockade in nonfailing trabeculae, especially at 2, 2.5, and 3 Hz (ANOVA, *P *<* *0.05; *P < *0.05). In nonfailing trabeculae, there was a ~3‐fold increase in normalized diastolic force from 0.5 to 3 Hz (*P *<* *0.05) (Fig. 4C). Normalized diastolic force was also increased in failing trabeculae upon SR blockade (ANOVA, *P *<* *0.05) (Fig. 4D). The increase in normalized diastolic force from 0.5 to 3 Hz did not quite reach statistical significance (*P = *0.05) although the increase in normalized diastolic force from 0.5 to 2.5 Hz did reach statistical significance (*P *<* *0.05). Failing trabeculae also exhibited a ~3‐fold increase in normalized diastolic force at 3 Hz compared to 0.5 Hz after SR blockade (*P *<* *0.05).

### SR blockade results in slower contraction kinetics

**Figure 5:**
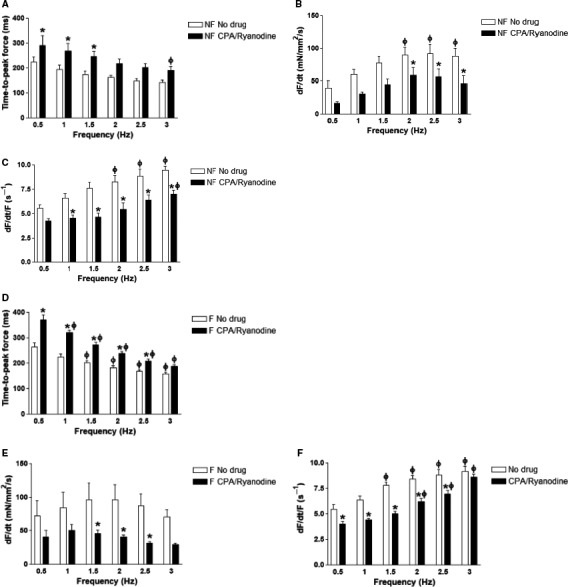
Contraction kinetics in nonfailing and failing trabeculae with and without SR blockade. (A) TTP in nonfailing trabeculae (*n* = 4) increased overall upon SR blockade with statistical significance at 0.5, 1, and 1.5 Hz. (B) Maximal rate of force development (dF/dt) in nonfailing trabeculae (*n* = 4) decreased overall upon SR blockade with statistical significance at 2, 2.5, and 3 Hz. (C) Maximal rate of force development normalized to developed force (dF/dt/F) in nonfailing trabeculae (*n* = 4) decreased overall upon SR blockade with statistical significance at 1, 1.5, 2, 2.5 Hz, and 3 Hz. (D) TTP in failing trabeculae (*n* = 5) increased overall upon SR blockade with statistical significance at 0.5, 1, 1.5, 2, and 2.5 Hz. (E) dF/dt in failing trabeculae (*n* = 5) decreased overall upon SR blockade with statistical significance at 1.5, 2, and 2.5 Hz. (F) dF/dt/F in failing trabeculae (*n* = 5) decreased overall upon SR blockade with statistical significance at 0.5, 1, 1.5, 2, and 2.5 Hz. *P <* *0.05 before versus after SR blockade after two‐way ANOVA with Bonferroni post‐hoc test. Φ indicates *P* < 0.05 versus corresponding 0.5 Hz parameter in the same group after two‐way ANOVA with Bonferroni post‐hoc test. SR, sarcoplasmic reticulum. TTP, Time‐to‐peak force.

Time‐to‐peak force (TTP) slowed down upon SR blockade in nonfailing and failing trabeculae (ANOVA, *P < *0.05) (Fig. 5A and D). For nonfailing trabeculae, the slowing of TTP was statistically significant at 0.5, 1, and 1.5 Hz (*P *<* *0.05). For failing trabeculae, statistical significance was reached at 0.5, 1, 1.5, 2, and 2.5 Hz (*P *<* *0.05). TTP was significantly decreased at 3 Hz compared to 0.5 Hz in nonfailing trabeculae following SR blockade (*P *<* *0.05). TTP was significantly decreased at 1.5, 2, 2.5, and 3 Hz compared to 0.5 Hz in failing trabeculae after SR blockade (*P *<* *0.05). In nonfailing and failing trabeculae, the maximal rate of force development (dF/dt) slowed down overall after SR blockade (ANOVA, *P *<* *0.05) (Fig. 5B). In nonfailing trabeculae, statistical significance was reached at 2, 2.5, and 3 Hz, and in failing trabeculae at 1.5, 2, and 2.5 Hz (*P *<* *0.05). Developed force and dF/dt are positively correlated, so we divided dF/dt by developed force to calculate a purely kinetic parameter, dF/dt/F, in the unit of (s^−1^). Upon SR blockade, dF/dt/F overall also slowed down in nonfailing and failing trabeculae (ANOVA, *P *<* *0.05) (Fig. 5C and F). For nonfailing trabeculae, statistical significance was reached at 1, 1.5, 2, 2.5, and 3 Hz (*P *<* *0.05). For failing trabeculae, statistical significance was reached at 0.5, 1, 1.5, 2, and 2.5 Hz (*P < *0.05). After SR blockade, dF/dt/F was increased from 0.5 Hz to 3 Hz in both nonfailing and failing trabeculae (*P *<* *0.05). Before SR blockade, dF/dt/F was increased at 1.5, 2, 2.5, and 3 Hz compared to 0.5 Hz in nonfailing trabeculae (*P *<* *0.05). After SR blockade, dF/dt/F was increased at 2, 2.5, and 3 Hz compared to 0.5 Hz in failing trabeculae (*P *<* *0.05).

### SR blockade does not affect early relaxation kinetics

**Figure 6:**
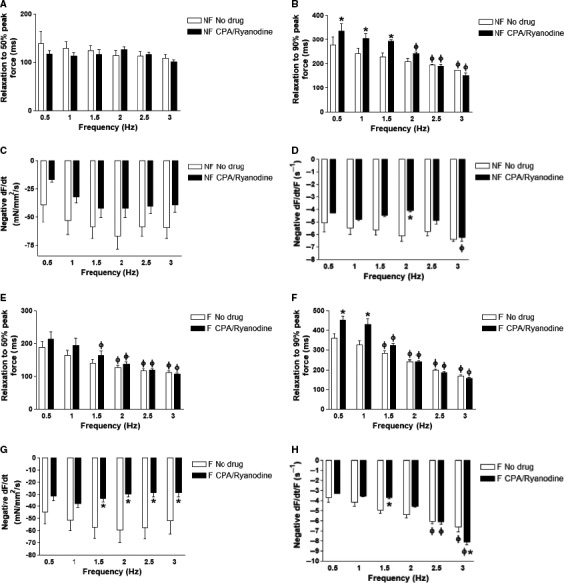
Relaxation kinetics in nonfailing and failing trabeculae with and without SR blockade. (A) Relaxation to 50% peak force time (RT50) in nonfailing trabeculae (*n* = 4) with SR blockade does not change significantly. (B) Relaxation to 90% peak force time (RT90) in nonfailing trabeculae (*n* = 4) overall was significantly slower upon SR blockade with statistical significance at 0.5, 1, and 1.5 Hz. (C) Maximal kinetic rate of relaxation (negative dF/dt) in nonfailing trabeculae (*n* = 4) overall was slower upon SR blockade but not at particular frequencies. (D) Maximal kinetic rate of relaxation normalized to developed force (negative dF/dt/F) overall was significantly slower upon SR blockade with statistical significance at 2 Hz. (E) RT50 in failing trabeculae (*n* = 5) was not significantly affected by SR blockade. (F) RT90 in failing trabeculae (*n* = 5) overall was significantly slower upon SR blockade with statistical significance at 0.5 Hz and 1 Hz. (G) Negative dF/dt in failing trabeculae (*n* = 5) overall was significantly slower upon SR blockade with statistical significance at 1.5, 2, 2.5, and 3 Hz. (H) Negative dF/dt/F in failing trabeculae (*n* = 5) overall did not significantly change upon SR blockade. However, negative dF/dt/F was slower upon SR blockade at 1.5 Hz and faster at 3 Hz. SR, sarcoplasmic reticulum.

Time‐to‐50% relaxation (RT50) did not significantly change due to SR blockade in nonfailing and failing trabeculae (ANOVA, *P = *0.35 and 0.08, respectively) (Fig. 6A and E). In nonfailing trabeculae, RT50 did not significantly shorten as frequency was increased. In failing trabeculae, RT50 did significantly shorten, compared to 0.5 Hz, at 1.5, 2, 2.5, and 3 Hz. After SR blockade, RT50 in failing trabeculae were shortened at 2, 2.5, and 3 Hz (*P < *0.05). However, time‐to‐90% relaxation (RT90) across the tested frequencies was significantly slower upon SR blockade in nonfailing and failing trabeculae (ANOVA, *P < *0.05) (Fig. 6B and F). In nonfailing trabeculae, statistical significance was reached at 0.5, 1, and 1.5 Hz (*P < *0.05). In failing trabeculae, statistical significance was reached at 0.5 Hz and 1 Hz (*P < *0.05). After SR blockade, RT90 in nonfailing trabeculae were significantly faster, compared to 0.5 Hz, at 2 Hz, 2.5, and 3 Hz (*P *<* *0.05). In failing trabeculae, RT90 was faster at 1.5, 2, 2.5, and 3 Hz with and without SR blockade (*P *<* *0.05). The maximal rate of force decay, negative dF/dt was slower in nonfailing trabeculae upon SR blockade but not at particular frequencies (ANOVA *P < *0.05) (Fig. 6C). In failing trabeculae, negative dF/dt was also slower upon SR blockade overall and particularly at 1.5, 2, 2.5, and 3 Hz (ANOVA *P < *0.05; *P < *0.05) (Fig. 6G). In nonfailing trabeculae, negative dF/dt/F was slower overall after SR blockade (ANOVA *P < *0.05) (Fig. 6D). At 2 Hz, negative dF/dt/F was significantly slower after SR blockade (*P < *0.05). In failing trabeculae, negative dF/dt/F overall was not different upon SR blockade (ANOVA, *P > *0.05) (Fig. 6H). At 1.5 Hz, negative dF/dt/F was significantly slower upon SR blockade (*P < *0.05). At 3 Hz, negative dF/dt/F was significantly faster upon SR blockade (*P < *0.05).

### SR blockade results in increased arrhythmogenicity in failing myocardium

**Figure 7:**
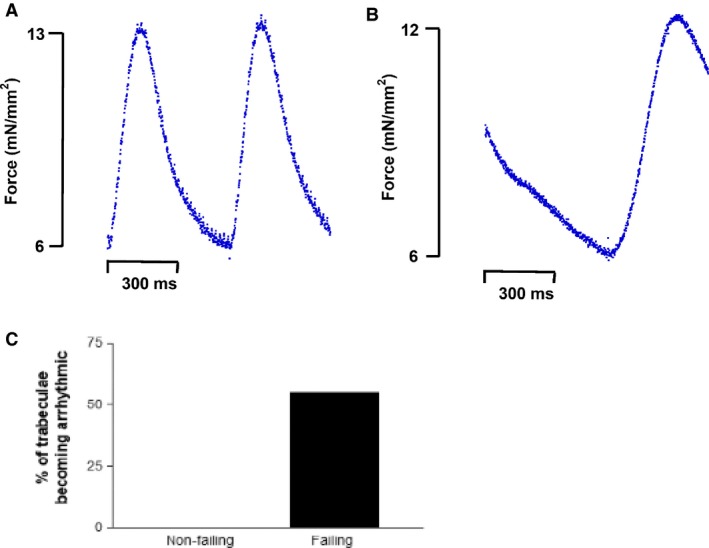
Example of a failing trabeculae becoming arrhythmic with SR blockade beating at 2 Hz. (A) Failing trabeculae contracting at 2 Hz without SR blockade (B) Failing trabeculae undergoing arrhythmia upon SR blockade (C) 55% of failing trabeculae (six of 11) became arrhythmic upon SR blockade compared to 0% for nonfailing trabeculae (0 of 4). SR, sarcoplasmic reticulum.

During our SR blockade experiments, we noticed that some of failing trabeculae became arrhythmic upon SR blockade. We went back to our data and found that six of 11 (55%) failing trabeculae became arrhythmic upon SR blockade at high frequencies (2–3 Hz). In contrast, 0 of 4 nonfailing trabeculae became arrhythmic upon SR blockade (Fig. 7). This result was not quite statistically significant per two‐sided Pearson chi‐Square analysis (*P *=* *0.057).

## Discussion

In this study, we show that intact right ventricular trabeculae from nonfailing and failing human hearts are able to sustain contractions at baseline (stimulated at 1 Hz frequency) as well as at lower and higher frequencies without functional SR calcium cycling. Upon SR blockade, trabeculae were able to generate similar amount of developed force (trending toward lower developed force although not statistically significant). However, the rate at which they were able to generate force was significantly slower. This suggests that the SR may not be necessary for force‐generation as much as be involved in the rate of force‐generation. During systole, the L‐type (Long) calcium channel very likely compensates for the lack of SR calcium release. During diastole, the sodium calcium exchanger (NCX) is likely responsible for compensating for sequestration of intracellular calcium via SERCA which allows the calcium concentration to decline to help the heart relax.

FFR is an important mechanism that allows the heart to modulate its cardiac output at various heart rates. One important finding from this study is that, in human cardiac trabeculae isolated from nonfailing and failing hearts, FFR is maintained even when the SR is no longer functional, showing that the SR is not necessary for FFR. Rather, our findings suggest other mechanisms such as changes in myofilament calcium sensitivity combined with calcium influx through L‐type calcium channel likely play a very significant role in regulation of the FFR. Based on previous studies (Pieske et al. 1996; Rossman et al. 2004; Chung et al. 2018), nonfailing trabeculae typically display a positive FFR, as developed force increases at increased stimulation frequencies. Failing trabeculae typically have a negative FFR as developed force decreases at increased stimulation frequencies. In addition, ‐dF/dt/F is overall slower at all physiologically relevant stimulation frequencies (0.5–3 Hz) in failing trabeculae (Chung et al. 2018).

In rabbits, a positive FFR was also preserved upon SR blockade, but the FFR was significantly less positive after SR blockade. (Monasky and Janssen 2009). In our study in human trabeculae, FFR actually became more positive upon SR blockade in nonfailing trabeculae and more negative in failing trabeculae. This is due to the fact that developed force before and after SR blockade was not significantly different at 1.5 and 2 Hz, frequencies where maximal developed force is typically reached (Milani‐Nejad et al. 2015). This suggests that the SR is not necessary for force development, as the L‐type calcium channel can provide the amount of calcium needed for proper contraction. In failing trabeculae, FFR became more negative at high frequencies upon SR blockade, but there was no significant difference in the absolute developed force. As in nonfailing trabeculae, it appears that the lack of SR‐mediated calcium release is compensated by other means of raising the intracellular calcium levels, such calcium entry through L‐type calcium channels.

Further, supporting the notion that L‐type calcium channel compensates for the SR is that parameters of contraction kinetics (TTP and dF/dt/F) were slower at all frequencies upon SR blockade, which is in agreement with previous work on rat and rabbit myocardium (Maier et al. 1998; Monasky and Janssen 2009). We believe that this phenomenon is due to the inability of the L‐type calcium channel to sufficiently compensate for the rate of intracellular calcium elevation, which is fast by the SR, but slower by L‐type calcium entry. Active developed forces are not significantly changed upon SR blockade. Therefore, L‐type calcium channel seems to be able to allow cardiomyocytes to take up the same or a similar amount of calcium as before the SR block. However, the rate at which this process occurs must be slower with the L‐type calcium channel becoming the sole driver of intracellular calcium increase. Upon SR blockade, L‐type calcium channels are not inactivated by increased local calcium concentration that primarily originates from the SR. This allows L‐type calcium channels to be open for a longer period of time to transport sufficient amount of calcium into the cytosol to compensate for the lack of SR calcium release. However, the rate at which this process occurs is believed to be slower than through SR calcium release, as the rate of calcium transport via L‐type calcium channel is about three times slower than the rate of calcium transport via ryanodine receptor (Bers 2001). This may explain why contraction kinetics are slower after SR blockade. At the moment, we do not have direct evidence that L‐type calcium channels are indeed the compensatory mechanism when the SR calcium release is inhibited. A possible future direction of our work may include utilization of calcium channel blockers such as verapamil to inhibit the function of L‐type calcium channels in human cardiac trabeculae. Another approach would be to measure the current through the L‐type calcium channels after SR blockade, but it is technically challenging to reliably perform this experiment in a human cardiomyocyte.

Upon SR blockade, diastolic force was increased in nonfailing and failing trabeculae, especially at high frequencies. We believe that this is most likely due to increased diastolic intracellular calcium. As a cardiac trabecula beats faster, it must also be able to sequester its intracellular calcium faster. Our data suggest that baseline calcium at diastole is elevated upon SR blockade at high frequencies. This might be due to the fact that cardiomyocytes having to rely on the sodium calcium exchanger (NCX) for calcium sequestration in the absence of a functioning SR. Normally, the NCX is only responsible for sequestration of approximately 30% of intracellular calcium in human myocardium, and our data imply that it may be oversaturated with calcium at high stimulation frequencies, which may lead to increased diastolic force. It has been previously shown that intracellular calcium content increases upon increased frequency (Eisner 2018). Interestingly, we also noted that nonfailing and failing trabeculae display the same increase in diastolic force, suggesting that the overall function of NCX is not drastically, if at all, different between nonfailing and failing trabeculae.

In this study, we found that early (first 50%) relaxation kinetics are not affected by SR blockade in both nonfailing and failing trabeculae. This suggests that the SR does not necessarily play a crucial role in the regulation of early relaxation kinetics. Myocardial relaxation is a complex property that involves not only calcium transient decline, but also myofilament calcium sensitivity, cross‐bridge cycling kinetics, and troponin‐C‐calcium kinetics (Biesiadecki et al. 2014), as well as is impacted by early ventricular volume/sarcomere length changes (Chung et al. 2016). In addition, our results suggest that these processes of early relaxation are not dysregulated in failing trabeculae, as RT50 was not affected by SR blockade in nonfailing and failing trabeculae.

However, when we considered the kinetics of the entire relaxation process (RT90), we noticed slowing of RT90 at lower frequencies, 0.5 Hz and 1 Hz, upon SR blockade. This was an interesting phenomenon that suggests that SR plays a significant role in the later 50% of relaxation at lower frequencies, but not at high frequencies. It is possible that the increased intracellular calcium concentration at high frequencies leads to altered electrochemical gradient in cardiomyocytes, to more favor efflux of calcium via NCX. Again, processes of relaxation other than SR seem to be unaffected by heart failure, as nonfailing and failing trabeculae display the same response to SR blockade. The maximal rate of force decay normalized to developed force, negative dF/dt/F, displayed an interesting behavior where, in nonfailing trabeculae, negative dF/dt/F slowed down at 2 Hz due to SR blockade. This is due to a high developed force at 2 Hz with relatively constant negative dF/dt. On the other hand, the magnitude of negative dF/dt/F measured after SR blockade at 3 Hz failing trabeculae was increased because of the decreased developed force with relatively constant negative dF/dt. Negative dF/dt/F trended to be slower upon SR blockade in nonfailing and failing trabeculae, but statistical significance was not reached.

During our experiments, six of 11 failing trabeculae became arrhythmic upon SR blockade, especially at high frequencies. However, 0 of 4 nonfailing trabeculae became arrhythmic. We hypothesize that the increased intracellular calcium leads to dysregulation of action potential by affecting sodium, potassium, and calcium currents.

In this study, we did not assess calcium transients. In human myocardium, this is technically more challenging than in animal myocardium, for several reasons, including preparation size, less uniform dye distribution, and significant loss of dye with rapid cooling contractures. In addition, the majority of the diseased hearts were from patients with a nonischemic etiology. Although in most of our past studies (Milani‐Nejad et al. 2015; Elnakish et al. 2017), non‐ischemic and ischemic muscles show a rather similar contractile profile, smaller quantitative differences may exist. Overall, since the impact of SR blockade is highly similar in failing and nonfailing tissue, impact of specific patient characteristics such as age or gender is likely not a major factor.

In this study, we have examined the role of SR calcium, which has been believed to be integral in cardiac contractile and relaxation as well as FFR via pharmacologic inhibition of the SR. We have found that trabeculae can actually contract and relax with little or no effect on their developed force and FFR without SR calcium. We believe that there must be calcium entry mechanisms (i.e., L‐type calcium channel or NCX) that allow the heart to compensate for the lack of functional SR and that further investigation into these mechanisms will enhance our understanding of cardiac contractile and relaxation properties in health and disease.

## Conflict of Interest

None decalred.
